# Graphene-based active slow surface plasmon polaritons

**DOI:** 10.1038/srep08443

**Published:** 2015-02-13

**Authors:** Hua Lu, Chao Zeng, Qiming Zhang, Xueming Liu, Md Muntasir Hossain, Philipp Reineck, Min Gu

**Affiliations:** 1 Centre for Micro-Photonics and CUDOS, Faculty of Science, Engineering and Technology, Swinburne University of Technology, Hawthorn, Victoria 3122, Australia; 2 Centre for Micro-Photonics, Faculty of Science, Engineering and Technology, Swinburne University of Technology, Hawthorn, Victoria 3122, Australia; 3 State Key Laboratory of Transient Optics and Photonics, Xi'an Institute of Optics and Precision Mechanics, Chinese Academy of Sciences, Xi'an 710119, China

## Abstract

Finding new ways to control and slow down the group velocity of light in media remains a major challenge in the field of optics. For the design of plasmonic slow light structures, graphene represents an attractive alternative to metals due to its strong field confinement, comparably low ohmic loss and versatile tunability. Here we propose a novel nanostructure consisting of a monolayer graphene on a silicon based graded grating structure. An external gate voltage is applied to graphene and silicon, which are separated by a spacer layer of silica. Theoretical and numerical results demonstrate that the structure exhibits an ultra-high slowdown factor above 450 for the propagation of surface plasmon polaritons (SPPs) excited in graphene, which also enables the spatially resolved trapping of light. Slowdown and trapping occur in the mid-infrared wavelength region within a bandwidth of ~2.1 μm and on a length scale less than 1/6 of the operating wavelength. The slowdown factor can be precisely tuned simply by adjusting the external gate voltage, offering a dynamic pathway for the release of trapped SPPs at room temperature. The presented results will enable the development of highly tunable optoelectronic devices such as plasmonic switches and buffers.

Optical signals in a medium of refractive index *n* will propagate with the group velocity *v_g_* = *c*/(*n* + *ω*d*n*/d*ω*), where *ω* and *c* are the angular frequency and speed of light in vacuum, respectively^1^. When the dispersion of the medium is negative (d*n*/d*ω* > 0), the light signal will be slowed down with respect to free propagation in the medium. Here, the slowdown factor or group index relative to free space is defined as *c/v_g_*^2^. This effect is commonly known as slow light. Potential applications of slow light are numerous and include optical data processing and communications, nonlinear optical enhancement and temporary storage of light^3^. To date, several mechanisms and materials have been explored to reduce the propagation speed of optical signals through a medium. They include the quantum interference effect (QIE)^4^, stimulated Brillouin scattering (SBS)^5^ and photonic crystals (PCs)^2^. Besides the comparably large size of devices, these approaches bear other inherent limitations, which hinder their practical application^6^. The operating bandwidth of slow light in QIE and SBS systems is very narrow due to the band-limited transparency windows^4^,^5^. The bandwidth in PCs can be extended, but at the cost of comparably low slowdown factors of 300^2^. New techniques to effectively control light on the micro/nano scale are indispensable for the miniaturization and development of efficient on-chip optical components. Surface plasmon polaritons (SPPs) are regarded as a promising physical mechanism to overcome the diffraction limit of light and to advance the miniaturization of devices^7^,^8^. In recent years, the generation, propagation, manipulation and detection of light, as well as light-matter interaction mediated by the excitation of SPPs in metal nanostructures have been investigated extensively^7^,^8^,^9^,^10^,^11^,^12^,^13^,^14^,^15^,^16^,^17^. Slow light in plasmonic structures has also attracted significant attention in the scientific community^18^,^19^,^20^,^21^,^22^,^23^. Sandtke *et al.* first observed a slowdown of a SPP mode in a metallic Bragg grating by a factor of 2^18^. To broaden the slow light bandwidth, Gan *et al*. proposed a graded grating structure to reduce the speed of light and realize a rainbow trapping effect on a metal surface^19^,^22^. However, the structure size is on the order of 20 μm, which is about 15 times larger than the operating wavelength. Moreover, the trapped waves could be released by temperature-tuning the refractive index of the materials. The releasing scheme is limited by the requirement of a dramatic change of device temperature.

Graphene, a two-dimensional monolayer of carbon atoms arranged in a honeycomb lattice, exhibits exceptional electronic, mechanical and optical properties^24^,^25^. In recent years, graphene has been introduced to the field of plasmonics^26^ and offers new ways to manipulate and confine light on the nanoscale. The intriguing properties of SPPs in graphene based structures were investigated in recent years^26^,^27^,^28^,^29^,^30^,^31^,^32^,^33^,^34^,^35^,^36^,^37^,^38^,^39^,^40^,^41^,^42^,^43^. For example, Vakil *et al.* found that graphene with spatially modulated conductivity patterns can be used as a one-atom-thick platform for transformation optics^27^. Wang *et al.* showed that graphene sheet arrays are capable of steering light efficiently through the SPP coupling between individual monolayers^30^. These results show that graphene plasmonics (GPs) offers a range of design possibilities for photonic applications. In particular, graphene based plasmonic structures exhibit relatively low dissipative loss, highly field confinement and dynamic tunability by external electromagnetic fields and chemical doping, making graphene a promising alternative to metal-based plasmonics^26^,^31^,^32^. Recently, the graphene structure has attracted attention for the generation of slow light effects^33^.

Here, we propose a novel structure composed of a monolayer graphene and a silicon based graded grating structure. Graphene and silicon are separated by a silica spacer layer and the surface conductivity of graphene can be tuned via an external gate voltage. We theoretically and numerically demonstrate that the SPP mode in graphene shows an exceptional slowdown factor above 450 with a broad bandwidth of ~2.1 μm in the mid-infrared optical region. Furthermore, the length of graphene structure is about 1/6 of the operating wavelength. Such a small size possesses a huge potential for the realization of on-chip optoelectronic components. The slowdown factor as well as the rainbow trapping performance of the system can be readily controlled via the external gate voltage, rendering the release of trapped waves feasible at room temperature.

## Results

### Model and analytical theory

**Figure 1:**
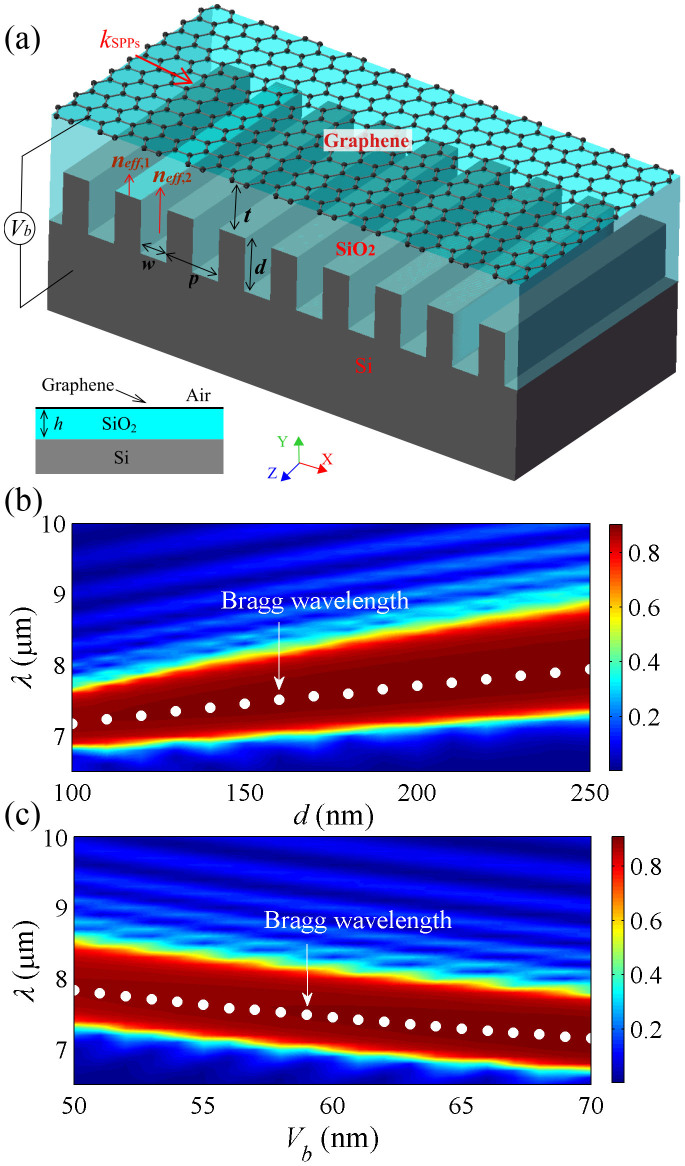
Schematic and reflectivity of the graphene Bragg grating. (a) Schematic illustration of the graphene-based plasmonic Bragg grating structure. The surface conductivity of the graphene monolayer on silica (SiO_2_) can be tuned through an external gate voltage (*V_b_*). The silicon (Si) substrate has a groove grating structure with period *p*, width *w* and depth *d*. *t* denotes the distance between the graphene and Si substrate. The inset shows a basic graphene-SiO_2_-Si structure with a SiO_2_ layer thickness *h*. (b) shows the reflectivity as a function of wavelength *λ* and grating groove depth *d* for a constant voltage *V_b_* = 60 V and (c) as a function of wavelength *λ* and applied gate voltage *V_b_* for a constant depth *d* = 150 nm. The other parameters are set as *t* = 100 nm, *p* = 2*w* = 40 nm, *T* = 300 K and *μ* = 20000 cm^2^V^−1^s^−1^. In the FEM simulations, the number of grating periods is 16 and SPP modes in graphene are excited from the left as indicated by the red arrow. The white circles represent the theoretical results for the Bragg grating operating wavelength obtained by solving Eq. (5).

To investigate photoelectric properties of graphene, we first discuss a simple multilayer structure consisting of a graphene monolayer on a doped silicon substrate, where the graphene and substrate are separated by a silica spacer layer with a thickness *h* (see the inset of Fig. 1(a)). A gate voltage is applied between the graphene sheet and Si substrate to set the doping level of graphene by the electric-field effect^34^. In the configuration, the light excitation in the mid-infrared spectral region occurs in the graphene and a transverse magnetic (TM) polarized SPP mode will propagate along the graphene monolayer. It is worth noting that the excitation of SPPs in graphene structures is exceptionally hard due to the momentum mismatch between the incident light and GPs. So far, many schemes have been exploited to excite the GPs, such as the prism coupling technique^36^, subwavelength silicon grating^37^ and resonant optical antennas^38^.

In the mid-infrared spectral region, the monolayer graphene can be characterized by a complex-valued surface conductivity *σ_g_*, which is quantitatively described by the Kubo formula^28^,^29^. The surface conductivity can be expressed as a sum of two terms: *σ_g_* = *σ*_intra_ + *σ*_inter_. The first term corresponds to the intraband electron-photon scattering and is given by
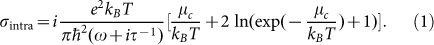


Here, *e* is the electron charge, *μ_c_* is the chemical potential, *ω* is photon frequency in vacuum, 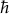 is the reduced Planck's constant, *k_B_* is Boltzmann's constant, *T* is the temperature and *τ* stands for the momentum relaxation time due to charge carrier scattering. The second term corresponds to the interband transition contribution. For 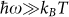 and |*μ_c_*| ≫ *k_B_T*, it can be approximately written as,
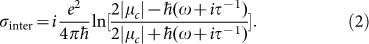


In graphene, *τ* depends on the carrier mobility *μ* and can be expressed as *τ* = *μμ_c_*/(*ev*_f_^2^). A previous report showed that *μ* = 230000 cm^2^V^−1^s^−1^ could be obtained experimentally in high-quality suspended graphene^44^. It was found that the carrier mobility of graphene on the SiO_2_ substrate could reach 40000 cm^2^V^−1^s^−1^ at room temperature^45^. To ensure the credibility of calculation results, a moderate mobility of *μ* = 20000 cm^2^V^−1^s^−1^ is chosen in our study^46^. The chemical potential can be expressed as 
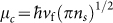
. Here, the Fermi velocity *v*_f_ is set as 10^6^ m/s^28^. The doping level of graphene (*n_s_*) shows a linear dependence on the external gate voltage described by the following equation^47^
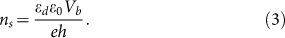


In this equation, *ε_d_* is the relative permittivity of SiO_2_ layer. Accordingly, *n_s_* can be set by the external voltage *V_b_* and the thickness *h* of SiO_2_ spacer, thereby controlling the chemical potential and surface conductivity of graphene. The dispersion relation of SPP modes in graphene can be derived from Maxwell's equations^48^
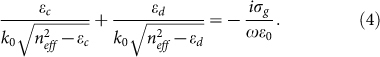


Here, *k*_0_ = 2π/*λ* is the free-space wave vector of light and *λ* is the operating wavelength in vacuum. In the mid-infrared region, the dielectric permittivities of SiO_2_ and air are assumed to be *ε_d_* = 3.9 and *ε_c_* = 1.0, respectively^49^. According to Eq. (4), the surface conductivity of graphene determines the effective refractive index *n_eff_* of the SPP mode, which is extremely sensitive to *h* and *V_b_*. Generally, the effective refractive index decreases with increasing *V_b_* due to the enlarged electric field. Thus, a larger *h* gives rise to a larger effective refractive index because the electric field on the graphene is smaller with a fixed voltage. In the mid-infrared region, the surface conductivity of graphene can be simplified into the Drude-like form^27^,^30^. From above equations, the real part of the effective refractive index could be approximated as 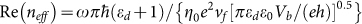. It shows that Re(*n_eff_*) is proportional to the square root of *h*, while inversely proportional to the square root of *V_b_*. The analysis is also verified by the calculation results (see Supplementary Information). It should be noted that for SiO_2_ spacer thicknesses above 100 nm, the influence of the Si substrate on the dispersion relation of the graphene SPP modes becomes negligible (see Supplementary Information). Thus, the above dispersion equation is obtained only considering the influence of the dielectric layers (air and SiO_2_) nearby the graphene.

### Graphene-based plasmonic Bragg grating

Figure 1(a) shows a graphene based Bragg grating structure, which consists of a graphene monolayer on a substrate made of Si. Both are separated by a dielectric layer of SiO_2_. The Si substrate has a groove grating with period *p*, width *w* and depth *d*. The thickness of SiO_2_ spacer (the distance between the graphene and Si substrate) is *t*. Here, the groove depth *d* is constant. In general, a Bragg grating is a periodic modulation of the refractive index of the propagation medium, which is designed to selectively reflect a specific wavelength of light and transmit the others. One common way to achieve this is by stacking two materials with different refractive indices^50^. A Bragg grating for the selective reflection or transmission of propagating SPPs in graphene is created by the periodic modulation of the graphene doping level and thus the effective refractive index of the graphene. This is achieved through the silicon grating underneath the graphene monolayer, which leads to a local modulation of *n_eff_* as described in the previous section.

According to the Bragg scattering condition, the wavelength at which SPP modes in graphene are reflected by the grating can be derived from
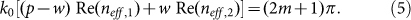


Here *n_eff_*_,1_ and *n_eff_*_,2_ represent the effective refractive indices of the SPP mode in the graphene with the two different areas above the silicon grating: *n_eff_*_,1_,where the separation between graphene and Si is low and *n_eff_*_,2_, where it is high (see Fig. 1(a)). *m* equals zero in the wavelength range of interest. The physical parameters are set as *p* = 40 nm, *w* = 20 nm, *t* = 100 nm, *V_b_* = 60 V and *T* = 300 K. The operating wavelength of this structure is calculated from Eq. (5) and the reflectivity determined numerically using the commercial finite-element method software COMSOL Multiphysics (see Methods section). Figures 1(b)–(c) show the reflectivity of the Bragg grating as a function of the grating groove depth *d* and gate voltage *V_b_* at different wavelengths. The calculation of Eq. (5) shows that the operating wavelengths of the Bragg grating red-shifts with increasing *d* (Fig. 1(b), white dots). This result is in excellent agreement with the numerical results shown in Fig. 1(b), where the red area indicates maximum reflectivity. The red-shift can be attributed to the increase of Re(*n_eff_*) with increasing grating groove depth *d*. Fig. 1(c) on the other hand demonstrates that the operating wavelength of the structure significantly blue-shifts with increasing gate voltage *V_b_*. Here, the blue-shift is caused by the change of Re(*n_eff_*), which decreases with increasing the applied voltage *V_b_*. From Eq. (5), *k*_0_ increases with the decrease of Re(*n_eff_*).

**Figure 2:**
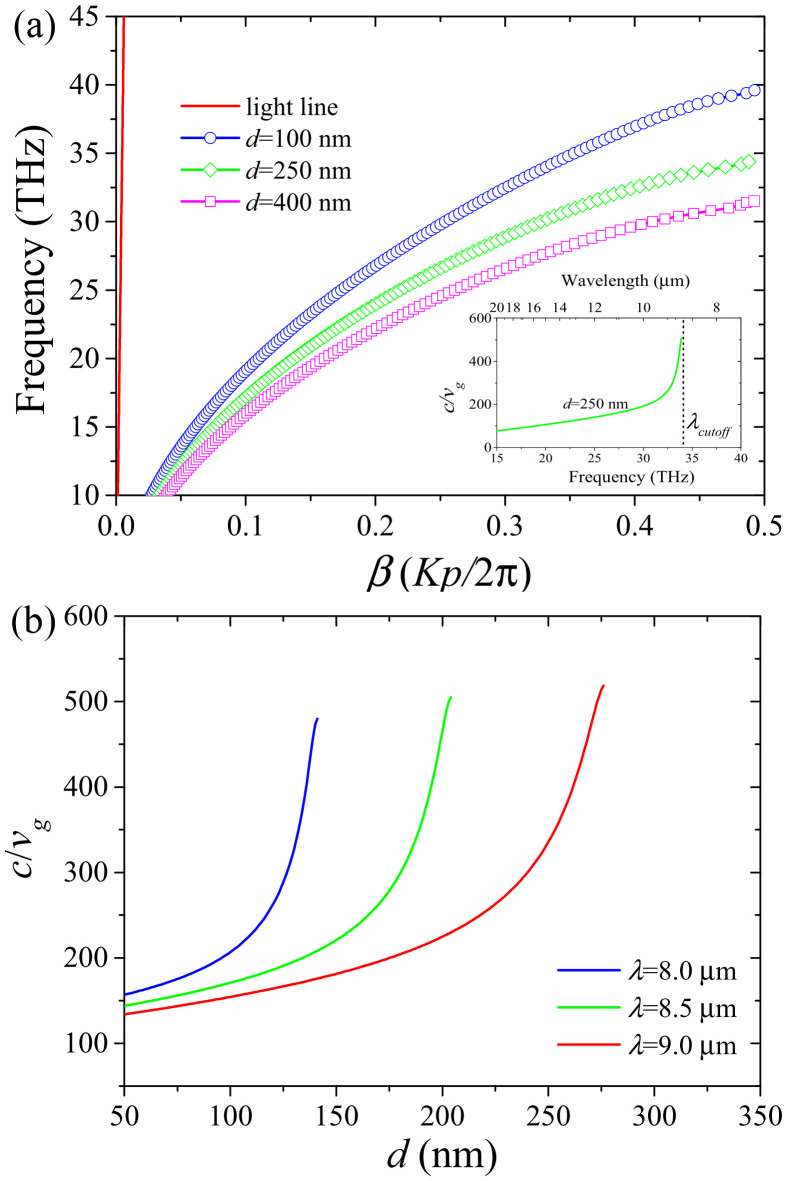
Dispersion curves and group indices of the graphene Bragg grating. (a) Dispersion curves for different substrate groove depth in the graphene based Bragg grating structure. *β* is the reduced propagation constant with a unit of *Kp*/2π. *K* is the Bloch vector of the SPP mode along the *x* axis and *p* is the period of the silica grating. The inset shows the group index (slowdown factor) as a function of frequency (and wavelength) for *d* = 250 nm. *λ_cutoff_* denotes the cutoff wavelength. (b) Group indices as a function of *d* for different operating wavelengths. In the calculations, *t* = 100 nm, *p* = 40 nm, *w* = 20 nm, *V_b_* = 60 V, *μ* = 20000 cm^2^V^−1^s^−1^ and *T* = 300 K.

Bragg gratings can be utilized to slow down the group velocity *v_g_* of light propagating in a medium^18^. This slowdown occurs at the edges of the Bragg grating operating region (red areas in Fig. 1). Therefore, the proposed Bragg grating structure is capable of slowing down the propagation of SPPs in the graphene monolayer. By using the characteristic equation (see Methods section), the dispersion curves for different groove depths are calculated and shown in Fig. 2(a). The results show that the cutoff frequency red-shifts with increasing groove depth *d*. The group velocity is obtained from the slope of the tangent of a dispersion curve at a given point (i.e., *v_g_* ≡ *∂ω*/*∂k*)^21^. The inset of Fig. 2(a) depicts the group index (*c*/*v_g_*) of the graphene Bragg structure as a function of frequency (or wavelength). It demonstrates that a light signal in the graphene layer propagates at velocities more than 450 times slower than the speed of light as its frequency approaches the cutoff frequency. The slowdown factor is limited by ohmic losses in graphene and can be further improved by changing the physical factors such as carrier mobilities in graphene (see Supplementary Information). In general, the ability of plasmonic structures based on graphene to slow down the propagation of light rivals their metal-based counterparts^18^,^22^,^51^. The slowdown factor is even 3 times larger than that of the reported graphene structure^33^. In agreement with the results presented in [19], we find that the slowdown factor strongly depends on the substrate groove depth at a given operating wavelength. The most efficient reduction of the group velocity of SPP modes in the structure presented here occurs when the groove depth of the silicon grating approaches the cutoff value of a given SPP mode wavelength (Fig. 2(b)). The groove depth required to achieve the most pronounced slowdown factor (or group index) increases with the operating wavelength.

### Graphene-based plasmonic graded grating

**Figure 3:**
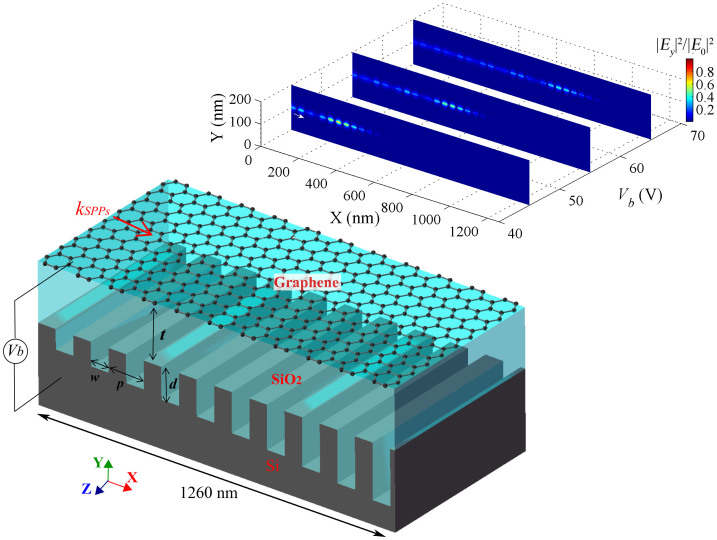
Schematic illustration of the graphene plasmonic graded grating structure. The groove depth *d* increases gradually along the direction of light propagation. *p*, *w* and *t* are the grating period, the groove width and the thickness of SiO_2_ spacer, respectively. The inset shows cross-sectional contour plots of the field distribution |*E_y_*|^2^ along the graphene at the wavelength of 9.0 μm for different gate voltages. |*E*_0_| is the maximum value of |*E_y_*|. In the simulation, the parameters are set as *t* = 100 nm, *p* = 2*w* = 40 nm, *T* = 300 K and *μ* = 20000 cm^2^V^−1^s^−1^. *d* increases linearly from 100 nm to 400 nm with an increment of 10 nm.

It is important to note that the graphene based Bragg grating structure can only be employed to extremely slow down the group velocity within a narrow spectral region close to the cutoff frequency (or wavelength). To broaden the spectral region within which light signal can be slowed down efficiently, a graded grating structure (instead of a normal grating in the previous section) is introduced as illustrated in Fig. 3. If the grade is small enough, the whole structure can be approximated as a sequence of gratings with uniform substrate groove depths^19^. Thus, the dispersion relation of the graphene based graded grating structure will change gradually in the direction of propagation of the SPPs (*x*-axis in Fig. 3). The group velocity of the SPP wave at a given wavelength will decrease with increasing groove depth as it propagates along the structure. As the SPP wave approaches the region, which corresponds to its cutoff frequency, its velocity is substantially decreased. Therefore, SPPs with different wavelengths can be trapped at distinct positions along the graphene monolayer, resulting in a large bandwidth.

To illustrate these effects in the graphene-based graded grating structure, the groove depth *d* is chosen to gradually increase from 100 nm to 400 nm along the *x*-axis, while pitch (*p* = 40 nm), width (*w* = 20 nm) and SiO_2_ thickness (*t* = 100 nm) remain constant. In order for the light trapping effect to occur, the grade gradient must satisfy the adiabaticity condition, which can in our case be expressed as *δ* = *∂k*^−1^/*∂x* ≪ 1^22^. In our structure, the groove depth increase per pitch is 10 nm, for which we obtain *δ* < 0.013 at the wavelength of 10 μm (see Supplementary Information for details). Therefore, the structure satisfies the adiabaticity condition. To investigate the SPP propagation and the so-called “rainbow” trapping effect in the proposed structure, FEM simulations were carried out. To intuitively observe the spatial separation of optical waves with different frequencies, the continuous waves are usually used as the incident sources^6^,^19^,^21^,^23^.

**Figure 4:**
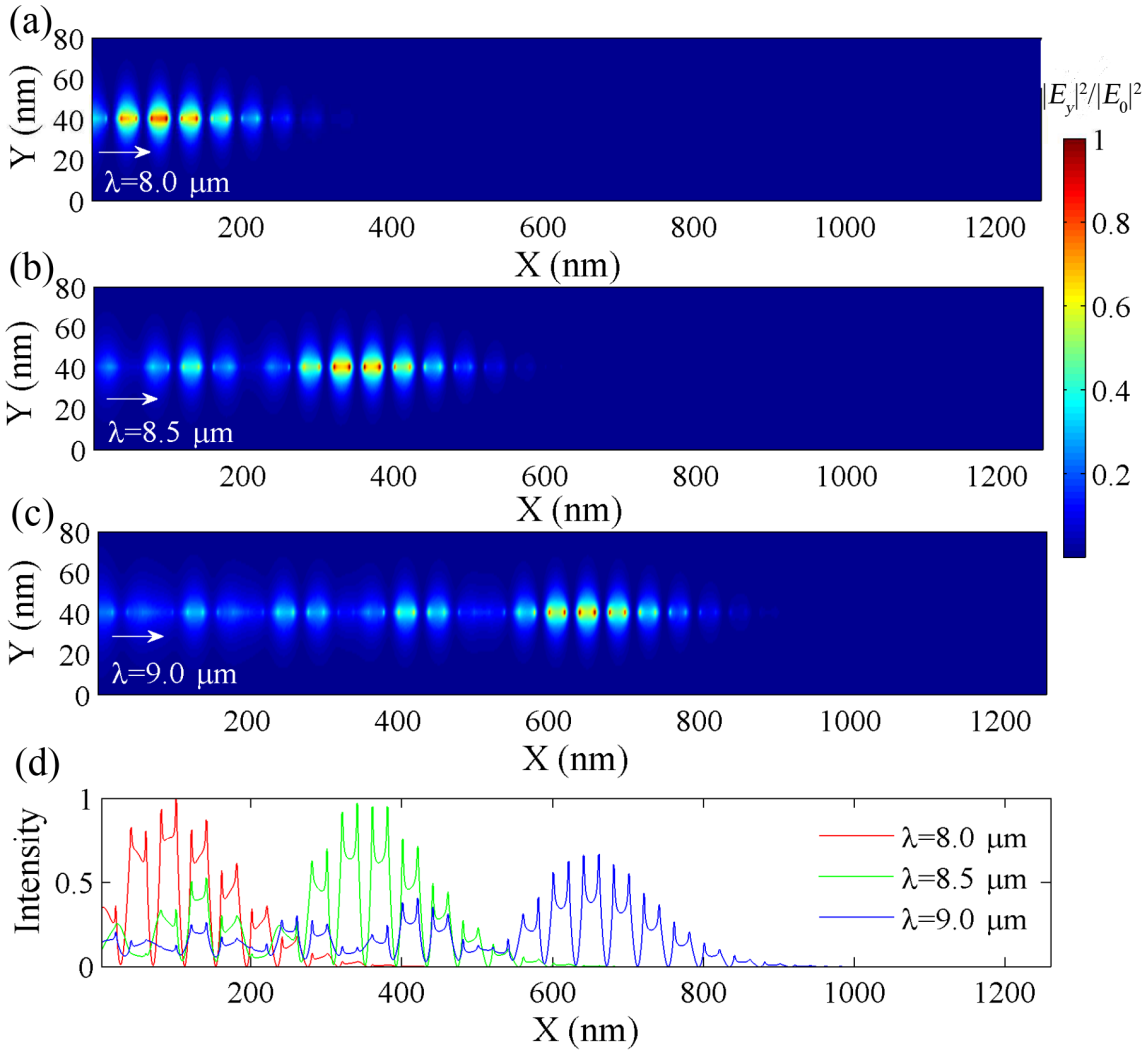
Electric fields of SPP waves in the graphene grated grating for different wavelengths. (a)–(c) show the normalized field distributions of |*E_y_*|^2^ in the *x*-*y* plane of the graphene graded grating structure in Fig. 3 for incident wavelengths of 8.0, 8.5 and 9.0 μm, respectively. (d) depicts the corresponding normalized field intensities 1 nm above the graphene sheet. In FEM simulations, the parameters are set as *t* = 100 nm, *p* = 2*w* = 40 nm, *V_b_* = 60 V, *μ* = 20000 cm^2^V^−1^s^−1^ and *T* = 300 K. The groove depth of the silicon grating under the monolayer graphene increases linearly from *d* = 100 nm to *d* = 400 nm. The increment per pitch for groove depth is 10 nm.

Figure 4 illustrates the electric field intensity (*|E_y_|*^2^) distribution in the *x*-*y* plane of the structure for incident wavelengths of 8.0, 8.5 and 9.0 μm. The wavelengths are chosen to be located between the previously calculated cutoff frequencies for the minimum and maximum groove depths in the structure of *d* = 100 and 400 nm. Fig. 4(d) reveals that the above light waves are trapped in the graphene monolayer at positions with groove depth values of 140, 205 and 275 nm. These positions respectively correspond to about 180, 420 and 700 nm along the *x*-axis, which is in excellent agreement with the results shown in Fig. 4. The SPP rainbow trapping effect occurs in the graphene structure. The length of graphene is about 6 times smaller than the operation wavelength, rendering an ultra-wide bandwidth of ~2.1 μm (from *λ* = 7.65 to *λ* = 9.73 μm) feasible. The bandwidth corresponding to ~8.5 THz is improved by a factor of 4 when comparing with the results in Ref. 33. The bandwidth can also be further broadened by increasing the number of substrate grooves. However, it should be noted that the SPP modes can never be completely trapped due to absorption losses in graphene, similar to those occurring in metal-based plasmonic waveguides^19^. The lifetime of the plasmonic modes in the monolayer graphene is estimated to be on the order of picoseconds (see Supplementary Information). To achieve perfect trapping of SPP modes in our structure, gain media could be introduced around the graphene monolayer to compensate dissipative losses. Thus, the group velocity *v_g_* can potentially be slowed down ever further and eventually approach zero.

**Figure 5:**
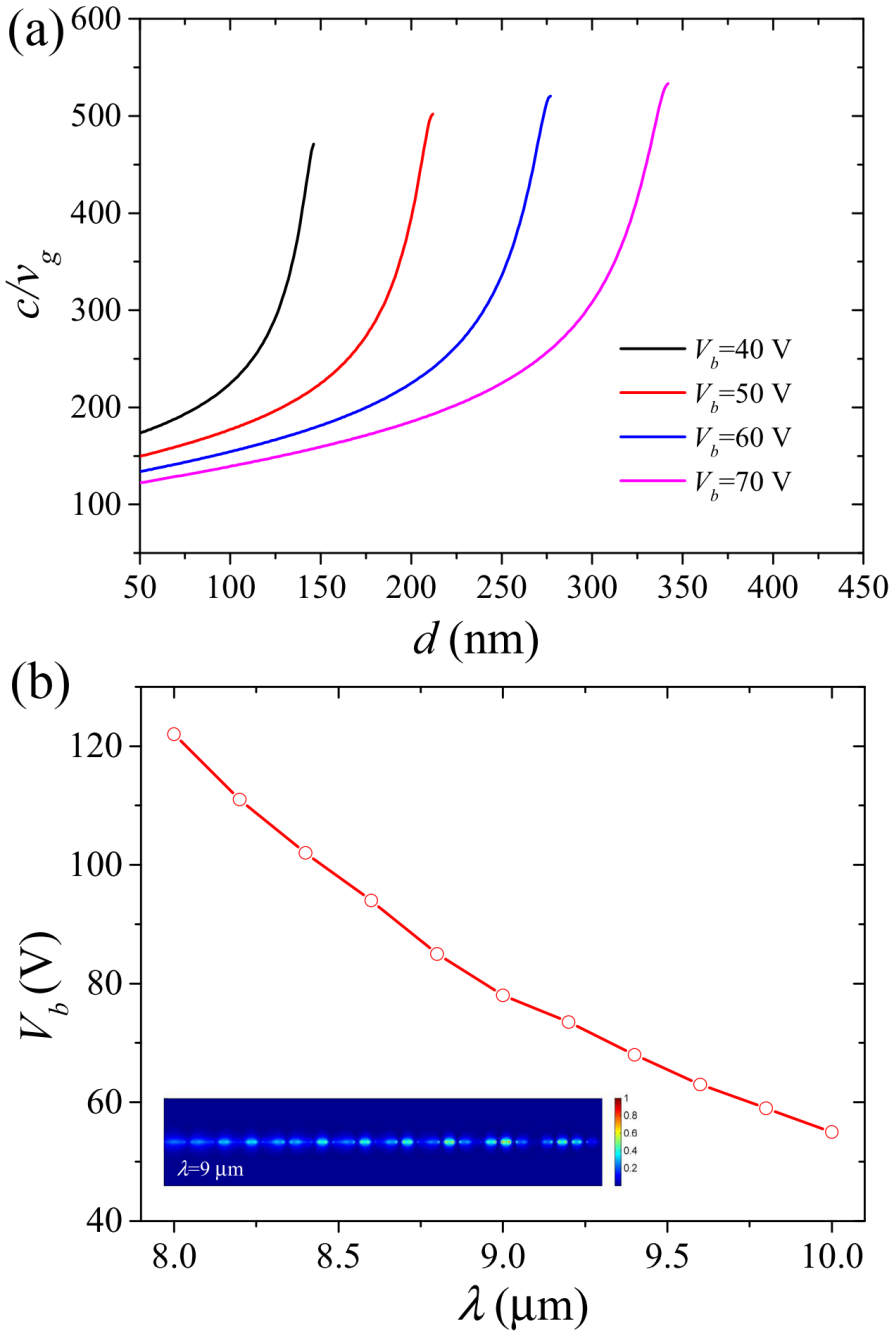
Voltage-controlled group indices and critical voltages for the release of trapped waves. (a) Group index as a function of substrate groove depth *d* for different gate voltages *V_b_* at 9.0 μm wavelength. (b) Theoretical critical gate voltages needed to release trapped waves as a function of wavelength at the position *x* = 1260 nm (output position). The other parameters are *t* = 100 nm, *p* = 40 nm, *w* = 20 nm, *μ* = 20000 cm^2^V^−1^s^−1^ and *T* = 300 K. The inset is the field distribution |*E_y_*|^2^ at the wavelength *λ* = 9.0 μm when *V_b_* = 85 V.

Another desired feature of a light tapping structure is the ability to release trapped waves in a controlled manner. It has been demonstrated that trapped waves in metal-based plasmonic waveguides can be released by introducing thermo-optic materials^19^. However, the bandwidth of the released light is limited by the comparably small refractive index variation induced by the thermo-optic materials. Furthermore, the temperature change may also affect the optical performance of the system. In the structure presented here, the effective refractive index of the monolayer graphene can be easily controlled by adjusting the external gate voltage, leading to an altered dispersion relation for the graphene graded grating structure (see Supplementary Information). This unique feature allows for the controlled release of plasmonic trapped waves in graphene with a broad bandwidth. As shown in Fig. 5(a), the group index of a SPP wave at each position of the graphene structure can be tuned effectively by changing the gate voltage *V_b_*. Moreover, a stepwise increase of gate voltage will move the location at which a given wave is trapped continuously along the direction of propagation, as illustrated in the inset of Fig. 3. Here, the parameters from the previous section are used (Fig. 4) and only the gate voltage is varied. Once a certain threshold voltage for a given trapped wave is reached, the wave will be able to propagate along the monolayer graphene and out of the graded grating zone. This critical voltage needed to release a given trapped wave has been calculated and is plotted as a function of wavelength in Fig. 5(b). For the wavelength of 9 μm, the field distribution (at *V_b_* = 85 V) is plotted in the inset of Fig. 5(b). It shows that the wave is able to propagate through the entire structure, while it was trapped in the middle of the structure for a gate voltage of 60 V (Fig. 4(c)). Thus, trapped waves are released as the gate voltage increases. This effect starts on the right side of the structure and progresses to the left with increasing voltage, i.e. waves trapped further left will also be released successively with increasing voltage.

## Discussion

In this report, a novel structure consisting of a graphene monolayer and a graded silicon grating, which is separated from the graphene by a silica spacer layer, has been proposed. It has been demonstrated that the graphene-based plasmonic structure is capable of slowing down the group velocity of mid-infrared light propagating in graphene by a factor of more than 450. Numerical simulations as well as theoretical calculations reveal that both the grating groove depth and the applied gate voltage can be employed to efficiently tune the effective refractive index of the graphene monolayer. This allows for an exceptional tunability of the system's slowdown, light trapping and light release characteristics. By using a graded grating, incident light of a given wavelength can be trapped at a specific position in the graphene structure. Thus, optical input signals with different wavelengths can be separated spatially and trapped at specific positions in the graphene monolayer. The operating spectral bandwidth of this effect is ~2.1 μm in our structure and the trapping occurs on a length scale of one micrometer only, which is less than 1/6 of the wavelength of the propagating light. In particular, increasing the applied gate voltage allows for a controlled and systematic release of the trapped light at room temperature.

The unique optical features of the graphene-based plasmonic structure combined with its simple tunability may pave the way towards the realization of nanoscale optoelectronic devices, in particular on-chip optical buffers and switches for signal processing and communications.

## Methods

The proposed graphene based structures are simulated using the finite-element method (FEM) software package COMSOL Multiphysics. In simulations, the graphene monolayer is represented by an ultrathin layer with a thickness of Δ = 1 nm^27^,^30^. Thus, the relative equivalent permittivity of graphene can be expressed as^27^
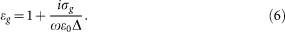


It should be noted that Δ is not the actual thickness of a graphene monolayer (~0.33 nm). It is found that deviations between results obtained for Δ = 1 nm and Δ = 0.33 nm could be negligible if the simulation mesh is chosen to be sufficiently fine^27^. We also find that the 2D simulations enable to accurately approximate the calculations of 3D graphene structures when the graphene width is large enough (many SPP wavelengths). Moreover, the 2D calculations could effectively save the computer memory and calculation time, which were broadly employed in plasmonic systems^19^,^20^,^21^,^23^,^38^,^52^.

To theoretically calculate the dispersion relation of the graphene-based grating structure, we employ the following characteristic equation^21^.
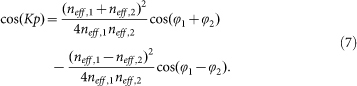


Here, *K* is the Bloch wave number of SPPs in the direction along *x* axis. *φ*_1_ = *k*_0_*n*_eff,1_(*p*-*w*) and *φ*_2_ = *k*_0_*n*_eff,2_*w* represent the plasmonic phases of the graphene zones with SiO_2_ thickness of *t* (non-groove parts) and *t* + *d* (groove parts), respectively.

## Author Contributions

H.L. conceived the idea, carried out the numerical simulation and wrote the manuscript text. C.Z., Z.Q.M., X.M.L., H.M.M., R.P. and M.G. discussed the design of the proposed structure and simulation results, as well as improved the manuscript presentation. All authors discussed the results and substantially contributed to the manuscript.

## Supplementary Material

Supplementary Information Supplementary Information
