# Supplementary material for: Graphene-based active slow surface plasmon polaritons
Source: Sci Rep. 2015 Feb 13;5:8443. doi: 10.1038/srep08443 (PMC4327412; doi:10.1038/srep08443)
Supplement: Supplementary Information [file srep08443-s1.doc]

**Supplementary Information**

Graphene-based active slow surface plasmon polaritons

**Hua Lu^1,2,3,*^, Chao Zeng^3^, Qiming Zhang^2^, Xueming Liu^3^, Md Muntasir Hossain^2^, Philipp Reineck^2^ and Min Gu^1,2,†^**

^1^Centre for Micro-Photonics and CUDOS, Faculty of Science, Engineering and Technology, Swinburne University of Technology, Hawthorn, Victoria 3122, Australia

^2^Centre for Micro-Photonics, Faculty of Science, Engineering and Technology, Swinburne University of Technology, Hawthorn, Victoria 3122, Australia

^3^State Key Laboratory of Transient Optics and Photonics, Xi’an Institute of Optics and Precision Mechanics, Chinese Academy of Sciences, Xi’an 710119, China

***^*^*** [*luhua@opt.ac.cn*](mailto:luhua@opt.ac.cn)

**^†^** *mgu@swin.edu.au*

1. **Dispersion characteristics of graphene SPPs**

As shown in the inset of Fig. 1(a) in the main manuscript, a monolayer graphene is attached on a SiO_2_ layer (*ε_d_*=3.9) with a thickness *h*. The dielectric on top of the graphene is set as air (*ε_c_*=1.0). The surface conductivity of graphene is controlled by the external voltage *V_b_*. By solving Eq. (1) to (4) in the manuscript, the evolutions of effective refractive index *n_eff_* as a function of *h* and *V_b_* can be obtained and are plotted in Fig. S1 for the 7-12 µm wavelength range. Both real and imaginary parts of *n_eff_* increase significantly with increasing spacer thickness *h* and show a pronounced decrease with increasing gate voltage *V_b_*.

**
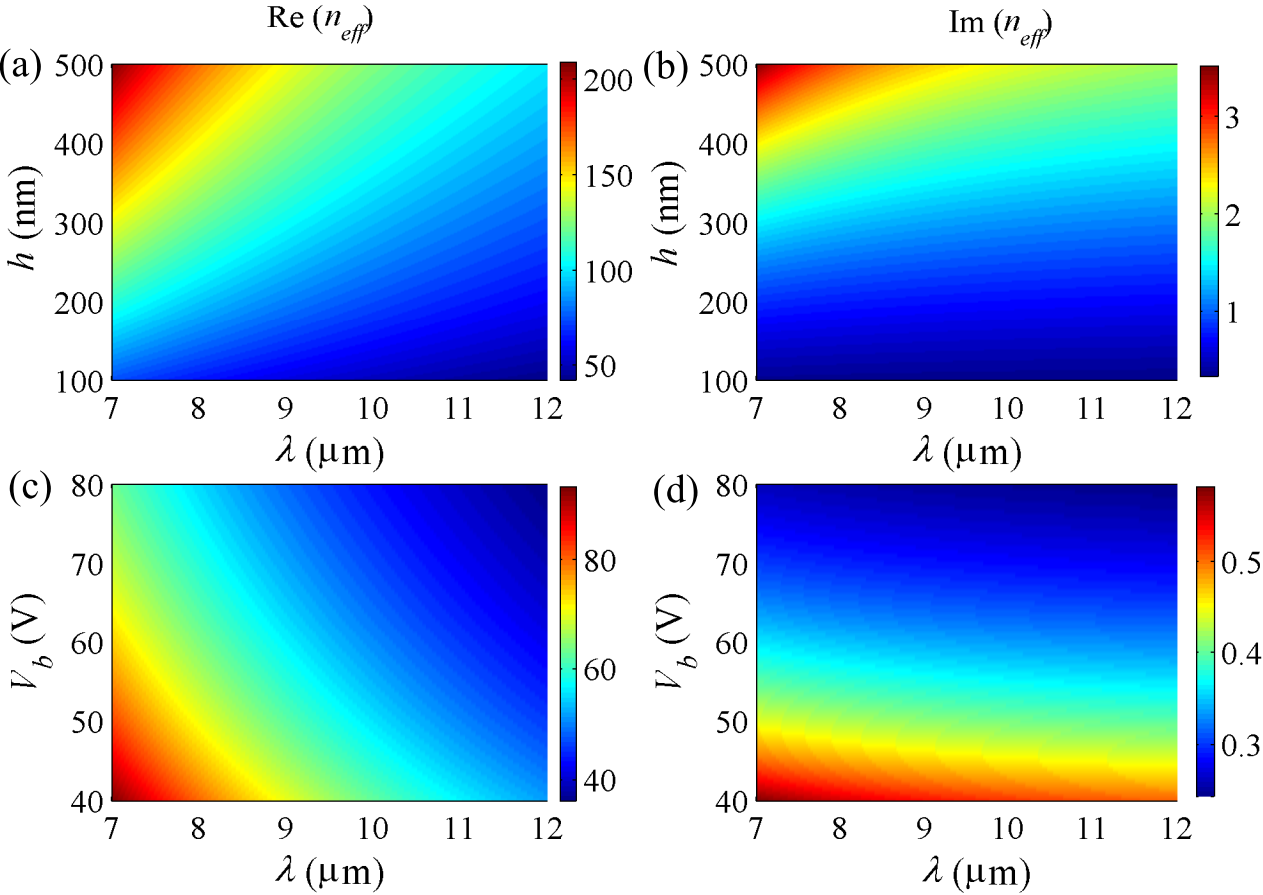
**

Figure S1. Real (a,c) and imaginary (b,d) parts of the effective refractive index (*n_eff_*) of SPP modes supported by the graphene monolayer as a function of wavelength *λ*. In (a) and (b), the gate voltage is constant (*V_b_* =60 V) and *n_eff_* is plotted as a function of SiO_2_ spacer thickness *h*. In (c) and (d), the thickness of SiO_2_ spacer is constant (*h*=100 nm) and the influence of the gate voltage *V_b_* on *n_eff_* is illustrated. Temperature and carrier mobility of graphene are *T*=300 K and *μ=*20000 cm^2^V^-1^s^-1^, respectively.

1. **Influence of Si substrate on the dispersion of graphene SPPs**

In the above section, the dependence of graphene plasmonic dispersion on *h* and *V_b_* has been discussed. However, the effect of the Si (*ε_s_*=11.7) substrate underneath SiO_2_ layer has been neglected due to the strong field confinement of SPP mode^S1^. To consider the influence of the Si substrate on the dispersion of graphene SPP modes, the dispersion relation can be determined by the following equations ^S2^,

$\exp(-2hk_{d})=\frac{(1+K_{a})(1+K_{b})}{(1-K_{a})(1-K_{b})}$ (S1)

$K_{a}=\frac{k_{d}}{k_{c}}\frac{\varepsilon_{c}}{\varepsilon_{d}}(1+\frac{i\sigma_{g}k_{c}}{\omega\varepsilon_{0}\varepsilon_{c}})$, $K_{b}=\frac{k_{d}}{k_{s}}\frac{\varepsilon_{s}}{\varepsilon_{d}}$ (S2)

Here, *k_c_*, *k_d_* and *k_s_* are the transverse propagation constants in the dielectric media air, SiO_2_ and Si, respectively. The propagation constants are relevant to the effective refractive index *n_eff_* and can be expressed as,

$k_{i}=k_{0}\sqrt{n_{eff}^{2}-\varepsilon_{i}},$ (*i*=*c*, *d* and *s*) (S3)

where *k*_0_=2π/*λ* is the free-space wave-vector of light. It is well-known that the plasmonic modes possess high confinement in graphene, thus the affect of Si substrate on the SPP mode will be weakened when the thickness of SiO_2_ increases^S2^. Here, we calculate the dispersion relation of GPs with a SiO_2_ thickness *h*=100 nm, which is the smallest value in our graphene grating structure. The obtained dispersion curves are compared with the results in Fig. S1. Figure S2 shows the effective refractive indices as a function of wavelength for external gate voltages *V_b_*=40 and 80 V. It is found that the dispersion curves considering the Si substrate almost overlap with the results ignoring the influence of substrate. It illustrates that the influence of Si substrate on the SPP dispersion can be neglected in our GP structures.

Figure S2. Effective refractive indices (*n_eff_*) of SPP modes in the graphene structure for *V_b_*=40 and 80 V when the thickness of SiO_2_ layer between graphene and Si substrate is *h*=100 nm. The circles represent the results obtained with considering Si substrate, and the lines stand for the results achieved without considering the influence of Si substrate.

**3. Dependence of group index on carrier mobility of graphene**

As mentioned before, the group index of slow light in metallic structure is dependent on the metal intrinsic loss^S3^. In the graphene structures, the intrinsic loss of graphene derives from the relaxation time *τ*, which can be expressed as *τ*=*μħ*(π*n_s_*)^0.5^/(*ev_f_*) ^S4^. The carrier density can be described as *n_s_*=*ε_d_ε*_0_*V_b_*/(*eh*) (see Eq. 3 in the manuscript). Thus, the relaxation time is dependent on the carrier mobility *μ*, external voltage *V_b_* and SiO_2_ spacer thickness *h*. According to the above equations, we can see that *τ* increases with *V_b_*. Thus, the propagation loss of SPP mode in the graphene will decrease, which agrees well with the result in Fig. S1(d). Meanwhile, a larger *h* gives rise to a smaller *τ*, inducing a larger propagation loss. The analysis is also verified by the result in Fig. S1(b). Moreover, the propagation loss will drop when the carrier mobility *μ* increases. As shown in Fig. S3, we calculate the group index nearby the cutoff wavelength as a function of *μ* in the graphene grating structure with the fixed parameters *t*=100 nm, *d*=250 nm and *V_b_*=60 V. It is found that the group index of graphene SPPs increases with the carrier mobility due to the decrease of graphene loss. With larger carrier mobilities, the slow light and rainbow trapping effects will obtain a further improvement.

Figure S3. Group indices near the cutoff wavelength as a function of carrier mobility *μ* in graphene structure with *t*=100 nm, *d*=250 nm, *p*=40 nm, *w*=20 nm, *V_b_*=60 V and *T*=300 K.

**4. Satisfaction of the adiabaticity condition**

The groove depth of substrate graded grating increases by a constant size from one period to next, we could evaluate the validity of the adiabatic or Wentzel-Kramers-Brillouin (WKB) approximation, also called the eikonal approximation using *δ=∂k*^-1^/*∂x* ^S5,S6^. For theoretical calculations, the detailed formula can be rewritten as,

$\delta\approx\frac{1/k_{1}-1/k_{2}}{p},$ (S4)

where *k*_1_ and *k*_2_ are the wave numbers in the adjacent grating units. *p* is the period of the grating. The criterion of applicability of the above WKB approximation is *δ*<<1^S6^. In our graphene structure, for example, the incremental step of *d* is set as 10 nm. As shown in Fig. S4, we obtain the value *δ*<0.013 at the wavelength of 10 μm for each selected *d* from 100 to 400 nm in the grating system. Thus, the 10 nm increment of groove depth reasonably meets the adiabaticity condition.

Figure S4. Adiabatic parameter, *δ*, for an incremental depth change of 10 nm as a function of groove depth in our graphene graded grating with *t*=100 nm, *w*=20 nm, *p*=40 nm, *V_g_*=60 V, *μ=*20000 cm^2^V^-1^s^-1^ and *T*=300 K.

**5. Lifetime of the SPP mode in the graphene grating**

We consider the lifetime of the plasmonic mode in graphene grating structure for the exploration of the usefulness in practical applications. The lifetime could be indirectly estimated by numerical simulations using the formula *τ_l_*=1/(*αv_g_*) ^S7^. Here *α* and *v_g_* are the propagation decay coefficient and group velocity of the SPP mode, respectively. *v_g_* can be determined from the slope of dispersion curves. 1/*α* corresponds to the decay length of the SPP mode in graphene, which can be calculated from the simulation results of the field intensity distribution. Figure S5 shows the intensity distribution 2 nm above the graphene sheet with the geometric parameters *d*=150 nm, *t*=100 nm, *p*=40 nm, *w*=20 nm, *V_b_*=60 V, *μ=*20000 cm^2^V^-1^s^-1^ and *T*=300 K. The operating wavelength is 10.5 μm. We can see that the peak intensity of the graphene plasmonic wave decrease along the graphene. The decay length is defined as the distance at which the intensity of the plasmonic wave decays to a factor of 1/e of its initial intensity. Thus, *α* can be extracted by fitting the peaks of field intensity. The large *α* derived from the strong absorption in the mid-infrared domain would indicate a shorter photon lifetime. As shown in Table S1, the derived lifetime of the SPP mode at the wavelength of 10.5 μm is a function of the substrate groove depth of graphene grating. As the SPP mode propagates in the graphene graded grating, the lifetime decreases slightly with increasing the groove depth. The plasmonic lifetime can approache ~0.69 ps when the groove depth is about 100 nm. This estimated value is larger than that in somewhat plasmonic structures^S8^. The reduction of graphene loss would be of help to trap light for a long period of time, which may contribute to the realization of nanoscale optical buffers.

Figure S5. Estimation of the propagation decay length at the wavelength of 10.5 μm along the graphene grating with *d*=150 nm. The intensity of field distribution |*E*|^2^ 2 nm above the graphene sheet is plotted. Here, *t*=100 nm, *p*=40 nm, *w*=20 nm, *V_b_*=60 V, *μ=*20000 cm^2^V^-1^s^-1^ and *T*=300 K.

Table S1. Estimation of the lifetime of plasmonic modes in the graphene grating structures with different groove depths.

| Operating wavelength *λ* (μm) | Groove depth *d* (nm) | 1/*α* (m) | *c/v_g_* | *τ_l_* (ps) |
| --- | --- | --- | --- | --- |
| 10.5 | 100 | 1.6319×10^-6^ | 126 | 0.6854 |
|  | 200 | 1.1567×10^-6^ | 156 | 0.6015 |
|  | 300 | 0.7772×10^-6^ | 193 | 0.5000 |
|  | 400 | 0.5056×10^-6^ | 250 | 0.4213 |

**6. Dispersion change of the graphene grating with the gate voltage**

To analyze the feasibility to release the plasmonic rainbow trapping, we theoretically calculate the change of dispersion relation of graphene grating with the different external bias voltages *V_b_*. As shown in Fig. S6, the cutoff frequency of the trapping light performs a blue-shift for increasing *V_b_*, and vice versa. This behavior provides an active control for the light trapping and releasing.

Figure S6. Dispersion curves for different external voltages *V_b_* in the graphene grating structure with *t*=100 nm, *d*=400 nm, *w*=20 nm, *p*=40 nm, *μ=*20000 cm^2^V^-1^s^-1^ and *T*=300 K.

**References**

S1. Chen, P. Y. & Alù, A. Atomically thin surface cloak using graphene monolayers. *ACS Nano* **5**, 5855-5863 (2011).

S2. He, X. Y. & Li, R. Comparison of graphene-based transverse magnetic and electric surface plasmon modes. *IEEE J. Sel. Top. Quant. Electron.* **20**, 62-67 (2014).

S3. Lu, H., Liu, X. & Mao, D. Plasmonic analog of electromagnetically induced transparency in multi-nanoresonator-coupled waveguide systems. *Phys. Rev. A* **85**, 053803 (2012).

S4. García de Abajo, F. J. Graphene plasmonics: challenges and opportunities. *ACS Photonics* **1**, 135-152 (2014).

S5. Gan, Q. *et al.* Experimental verification of the rainbow trapping effect in adiabatic plasmonic gratings. *Proc. Natl. Acad. Sci.* **108**, 5169-5173(2011).

S6. Durach, M., Rusina, A., Stockman, M. & Nelson, K. Toward full spatiotemporal control on the nanoscale. *Nano Lett.* **7**, 3145-3149 (2007).

S7. Gan, Q., Ding, Y. & Bartoli, F. ‘Rainbow’ trapping and releasing at telecommunication wavelengths. *Phys. Rev. Lett.* **102**, 056801 (2009).

S8. Vengurlekar, A. S., Gopal, A. & Ishihara, T. Femtosecond pulse distortion at surface plasmon resonances in a plasmonic crystal: Effect of surface plasmon lifetime. *Appl. Phys. Lett.* **89**, 181927 (2006).
